# Hypofunction of Circulating Endothelial Progenitor Cells and Aggravated Severity in Elderly Male Patients With Non-ST Segment Elevation Myocardial Infarction: Its Association With Systemic Inflammation

**DOI:** 10.3389/fcvm.2021.687590

**Published:** 2021-06-17

**Authors:** Lijin Zeng, Cong Zhang, Yuanting Zhu, Zhihao Liu, Gexiu Liu, Bin Zhang, Chang Tu, Zhen Yang

**Affiliations:** ^1^Department of Emergency, The First Affiliated Hospital, Sun Yat-sen University, Guangzhou, China; ^2^Department of Cardiology, The First Affiliated Hospital, Sun Yat-sen University, Guangzhou, China; ^3^National Health Commission (NHC) Key Laboratory on Assisted Circulation, Sun Yat-sen University, Guangzhou, China; ^4^School of Basic Medicine and Public Health Medicine, Institute for Hematology, Jinan University, Guangzhou, China; ^5^Department of Cardiovascular Disease, Jiangmen Central Hospital, Affiliated Jiangmen Hospital of Sun Yat-sen University, Jiangmen, China; ^6^Clinical Experimental Center, Jiangmen Central Hospital, Affiliated Jiangmen Hospital of Sun Yat-sen University, Jiangmen, China; ^7^Department of Cardiovascular Disease, The Third People's Hospital of Dongguan, Dongguan, China

**Keywords:** endothelial progenitor cells, aging, GRACE risk score, non-ST segment elevation myocardial infarction, interleukin-17

## Abstract

**Background:** Aging patients easily suffer from non-ST segment elevation myocardial infarction (NSTEMI). Our previous studies revealed declined function of endothelial progenitor cells (EPCs) in the elderly. However, the impact of aging on EPC function and severity in male NSTEMI patients and its possible mechanism is unclear until now.

**Methods:** We measured the circulating EPC function including migration, proliferation, and adhesion in aging or young male patients with NSTEMI. The GRACE and TIMI risk score were evaluated. Plasma levels of interleukin-6 (IL-6) and interleukin-17 (IL-17) were also detected in all patients.

**Results:** Compared with the young group, the old male patients with NSTEMI had higher GRACE score and TIMI score and decreased function of circulating EPCs. EPC function was negatively correlated with GRACE score and TIMI score. IL-6 and IL-17 level were higher in the old group than those in the young group. There was a significant negative correlation between EPC function and IL-6 or IL-17. Moreover, IL-6 and IL-17 positively correlated with GRACE and TIMI score. Age was positively related with GRACE or TIMI score and plasma level of IL-6 or IL-17, but inversely correlated with EPC function.

**Conclusions:** The current study firstly illustrates that the age-related decrement in EPC function is related to the severity of NSTEMI in male patients, which may be connected with systemic inflammation. These findings provide novel insights into the pathogenetic mechanism and intervention target of aging NSTEMI.

## Introduction

Aging which can cause organ function decline is an important independent predictor in cardiovascular disease (1, 2). The incidence and mortality in elderly patients with acute coronary syndrome (ACS) significantly increased when compared to young patients with ACS (3). GRACE and TIMI score are pivotal protocols to evaluate the severity and prognosis of NSTEMI in the clinic (4, 5). Nevertheless, the influence of aging on GRACE and TIMI score in NSTEMI remains notx fully understood.

Circulating endothelial progenitor cells (EPCs) is an important repair approach for vascular endothelial injury (6). Our previous investigation illuminated that aging led to mitigating EPC function via the CXCR4 pathway (7). It is reported that the function of late EPCs is subdued in elderly patients with acute myocardial infarction (AMI) (8), but other investigators found that the number of circulating CD34+CD133+ progenitor cells is not related to age after AMI (9). Because there is a great distinction in pathogenesis between ST-segment elevation myocardial infarction (STEMI) and NSTEMI, the effect of aging on EPC function in NSTEMI patients and its underlying mechanism are still unclear.

Interleukin-6 (IL-6) and interleukin-17 (IL-17) are important pro-inflammatory factors. The increased IL-6 can stimulate the differentiation of Th17 CD4 + T cells during inflammation, which leads to increment of IL-17 (10). It is reported that elevated IL-6 is found in acute myocardial infarction and related to EPC hypofunction (11). Accumulating evidence indicates that IL-6 and IL-17 increase with aging (12, 13). Base on the previous studies, we hypothesized that the attenuated circulating EPC quality may be related to exacerbated severity in aging male patients with NSTEMI, which may associate with the increment in plasma IL-6 and IL-17 levels. To test these hypotheses, we evaluated EPC function in young and aging male NSTEMI patients, compared their IL-17 and IL-6 levels, and analyzed the relationship between EPC function, severity of NSTEMI and inflammatory cytokines.

## Methods

### Characteristics of Patients

The NSTEMI diagnosis criteria was defined by acute chest pain, electrocardiogram and elevated cardiac Troponin T. 41 male NSTEMI patients were enrolled in the study, and their age was >18 years. All patients were classified into two groups: young group (<65 years) and aging group (≥65 years). Table 1 showed the basic characteristics of enrolled patients. Venous blood samples were collected to evaluate EPC function, plasma IL-17, and IL-6 levels.

**Table 1:** Clinical and biochemical characteristics in young and old-aged male NSTEMI.

**Characteristics**	**Young group (*n* = 20)**	**Old-aged group (*n* = 21)**
Age (years)	44.0 ± 7.4	77.2 ± 7.8^**#**^
Heart rate (time/min)	79.0 (71.3, 93.3)	75.0 (68.5, 82.5)
Systolic BP (mmHg)	131.6 ± 18.3	129.9 ± 22.7
Diastolic BP (mmHg)	80.0 (72.3, 94.5)	72.0 (68.5, 82.5)
BMI (kg/cm^2^)	25.7 ± 4.0	23.8 ± 3.8
Glucose (mmol/L)	5.8 (4.9, 11.2)	6.4 (5.6, 8.0)
Cr (mmol/L)	77.5 (60.7, 95.2)	82.4 (72.3, 120.9)
TC (mmol/L)	5.3 ± 1.5	4.5 ± 1.6
TG (mmol/L)	1.9 (1.4, 4.3)	1.3 (1.0, 2.5)
HDL (mmol/L)	1.1 (0.9, 1.2)	1.2 (1.0, 1.3)
LDL (mmol/L)	3.5 ± 1.2	2.9 ± 1.2
GRACE score	110.0 (100.3, 133.3)	179.0 (162.5, 196.0)^**#**^
TIMI score	3.0 (3.0, 3.0)	4.0 (3.0, 5.0)^**#**^

*BMI, body mass index; BP, blood pressure; Cr, serum creatinine; TC, total cholesterol; TG, triglyceride; HDL, high density lipoprotein; LDL, low-density lipoprotein*.

*Data which are normal distribution are given as mean ± SD and those which are abnormal distribution are given as median (25, 75%)*.

# *P < 0.05 vs. Young group*.

### Calculation of GRACE Risk Score

Calculation of the GRACE risk score was performed on the basis of each individual predictive factor and was calculated online (www.outcomes.org/grace).

### Calculation of TIMI Risk Score

The TIMI score was calculated as previous study described (4).

### EPC Function Assay

EPC function including migration, adhesion, and proliferation were evaluated according to previous investigations (14–17).

### Detection of IL-17 and IL-6

Plasma IL-17 and IL-6 levels have been tested by ELISA kit (R&D Systems, Minneapolis, USA) according to related studies (18, 19).

### Analysis of Statistics

Analysis of statistics was performed using the program SPSS (version 23.0; SPSS Inc., Chicago, Illinois). Comparisons between two groups were done with Student's *t*-test for the data with normal distribution while Mann–Whitney *U-*test was used for the data with abnormal distribution. Pearson test was performed for variables with normal distribution and Spearman test was used for abnormal distribution variables to evaluate the correlation. Values of *P* < 0.05 were considered as statistical significance.

## Results

### Baseline Characteristics

Baseline characteristics and laboratory findings of the young and old-aged groups were listed in Table 1. No significant difference of heart rate, blood pressure and BMI were found in the two groups (*P* > 0.05). The laboratory findings in plasma glucose, creatine, cholesterol, triglycerides, HDL, and LDL cholesterol were all similar between the two groups (*P* > 0.05). GRACE and TIMI score were remarkably higher in the old-aged group than the young (*P* < 0.05).

### EPC Function Between Young and Old-Aged Male Patients

**Figure 1:**
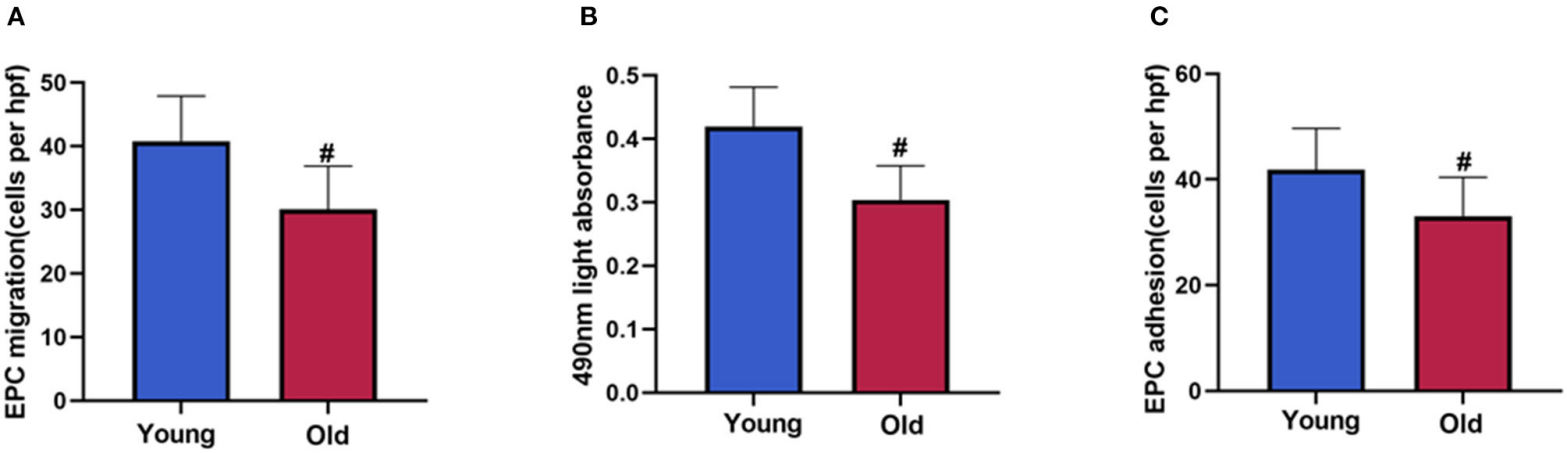
EPC function between young and old-aged group. The circulating EPC migration **(A)**, proliferation **(B)**, adhesion **(C)** decreased in old-aged patients with NSTEMI compared with young patients. Data are given as mean ± SD. ^#^*P* < 0.05 vs. young group.

Patients in the old-aged group presented decreased migration, proliferation and adhesion of circulating EPCs compared to the young group (Figures 1A–C, *P* < 0.05).

### Correlation Between the Severity of NSTEMI and EPC Function

**Figure 2:**
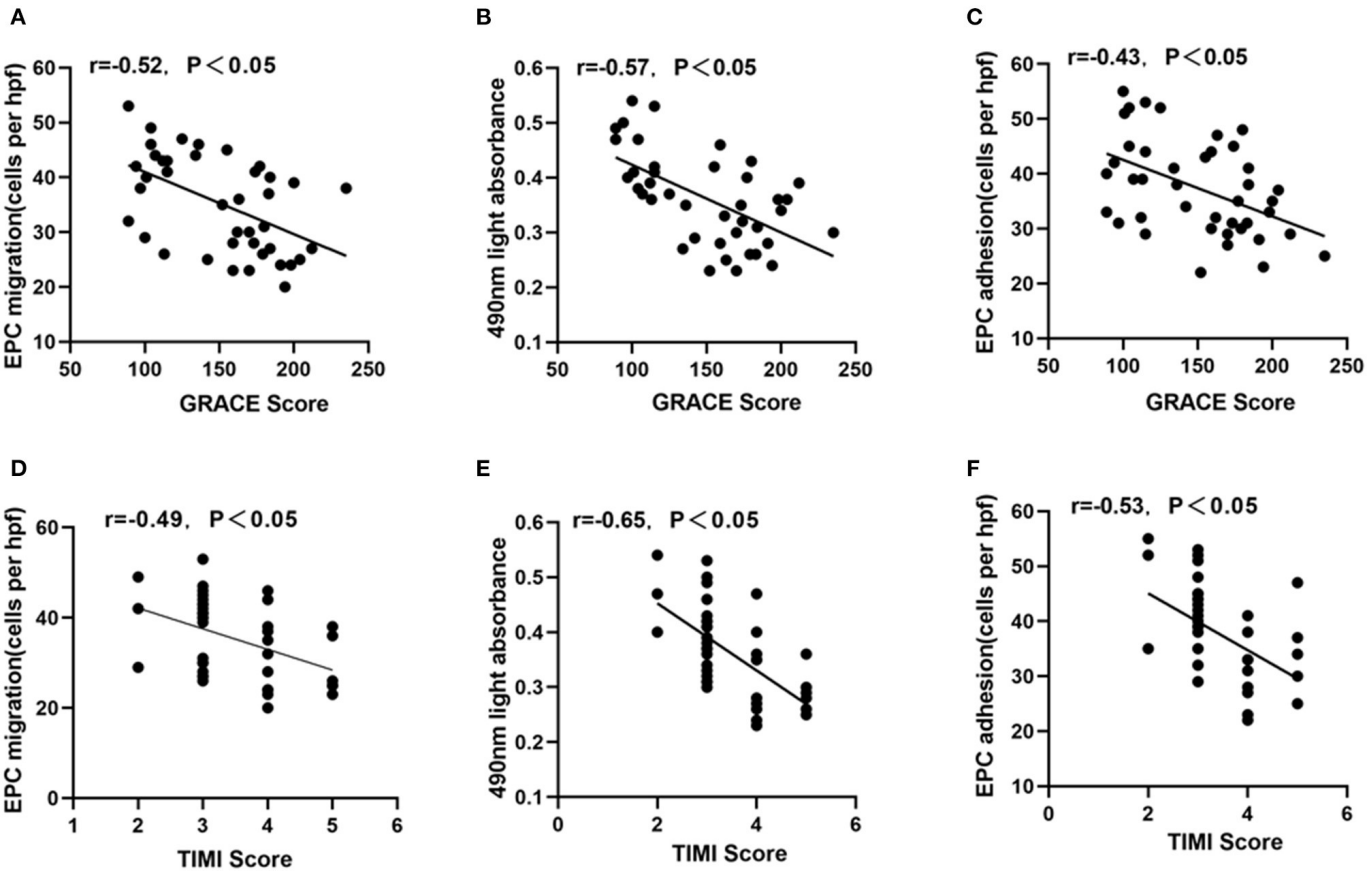
Correlation between GRACE or TIMI score and EPC function. GRACE score was negatively correlated with EPC migration **(A)**, proliferation **(B)**, adhesion **(C)**. Similarly, TIMI score was also inversely related with EPC migration **(D)**, proliferation **(E)**, and adhesion **(F)**.

Significant negative correlations between GRACE score and EPC migration, proliferation or adhesion were observed in male NSTEMI patients (Figures 2A–C, *P* < 0.05). The similar relationships were also found between TIMI score and EPC function (Figures 2D–F, *P* < 0.05).

### Correlation Between Inflammatory Cytokines and Circulating EPC Function or GRACE/TIMI Risk Score

**Figure 3:**
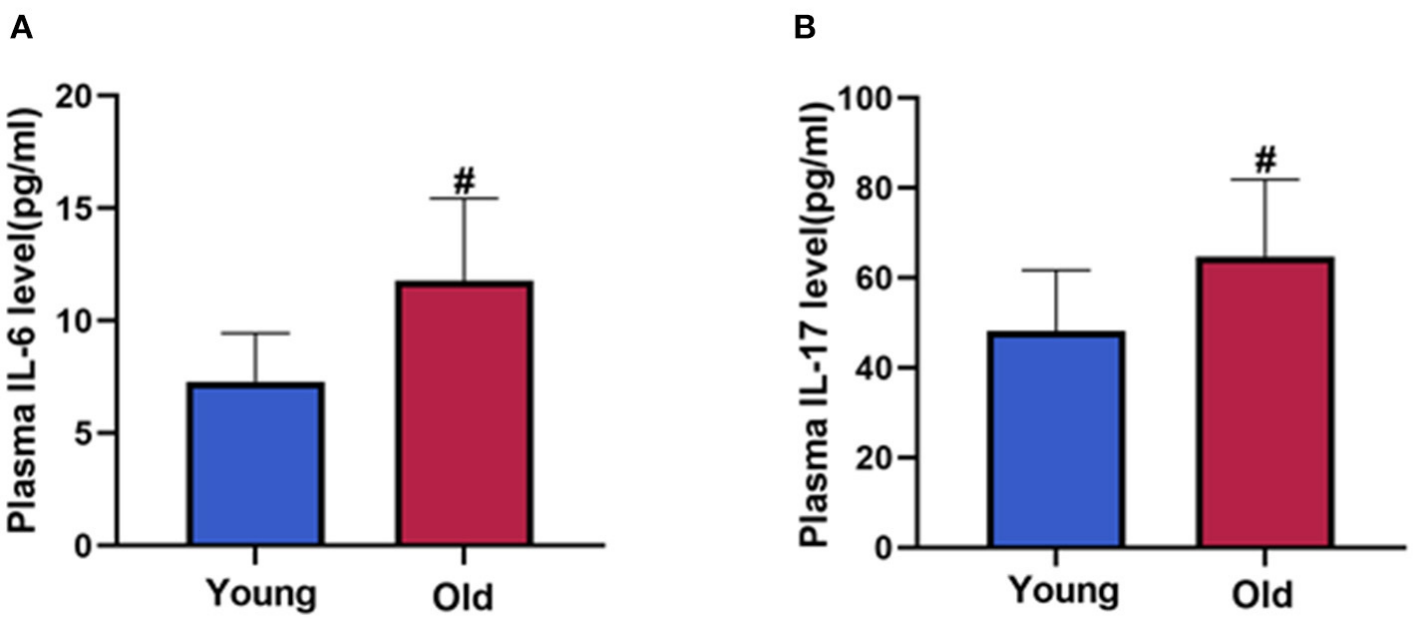
IL-6 and IL-17 level between young and old-aged group. Patients in old-aged group had higher levels of IL-6 **(A)** and IL-17 **(B)** than young patients. Data are given as mean ± SD. ^#^*P* < 0.05 vs. young group.

**Figure 4:**
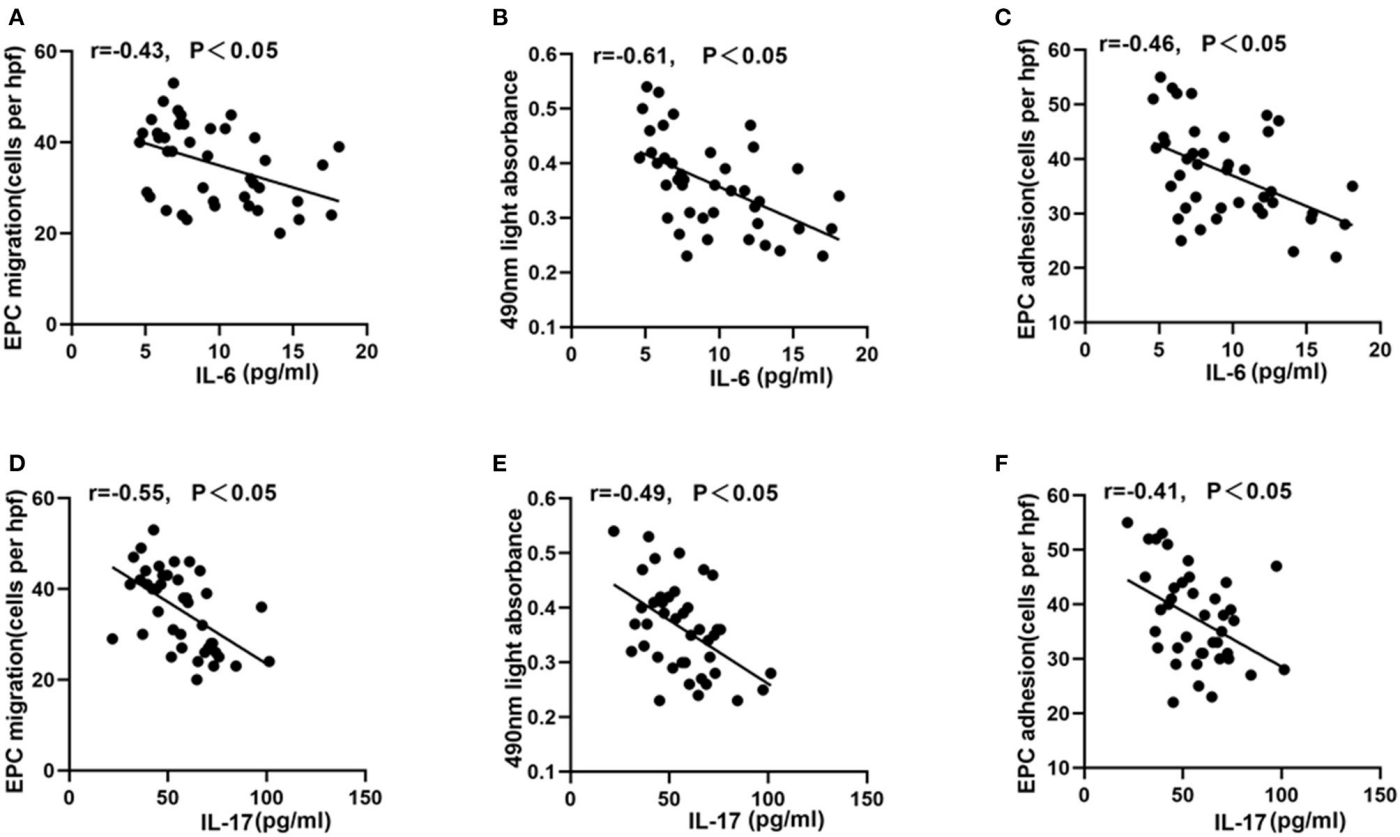
Correlation between EPC function and IL-6 or IL-17. IL-6 **(A–C)** and IL-17 **(D–F)** were negatively correlated with EPC migration, proliferation, and adhesion.

**Figure 5:**
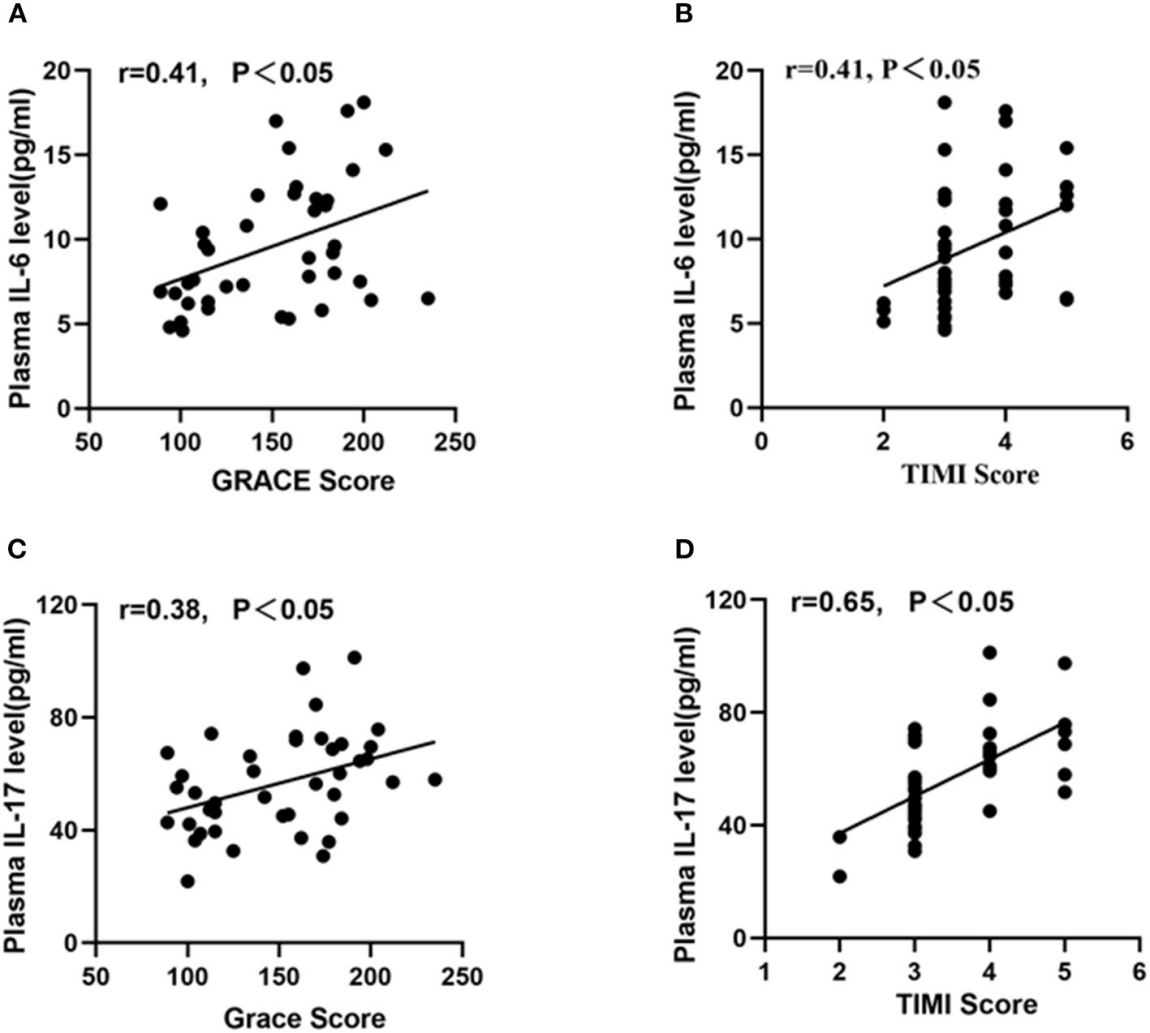
Correlation between inflammatory cytokines and GRACE or TIMI score. Both IL-6 **(A,B)** and IL-17 **(C,D)** were positively correlated with GRACE score and TIMI score.

As shown in Figures 3A,B, the plasma levels of IL-17 or IL-6 in young patients were lower than those in old-aged patients (*P* < 0.05). In addition, IL-17 and IL-6 were negatively correlated with the migration, proliferation, adhesion of circulating EPCs (Figure 4, *P* < 0.05). In contrast, IL-17 and IL-6 were positively correlated with GRACE and TIMI score (Figure 5, *P* < 0.05).

### Correlation Between Age and GRACE/TIMI Risk Score, Inflammatory Cytokines, and EPC Function

**Figure 6:**
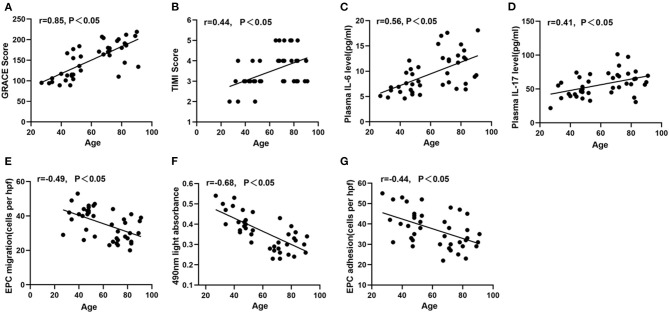
Correlation between age and GRACE or TIMI score or inflammatory cytokines or circulating EPC function. Age was positively correlated with GRACE score **(A)**, TIMI score **(B)**, IL-6 **(C)**, IL-17 **(D)**. Age was negatively correlated with EPC migration **(E)**, proliferation **(F)**, and adhesion **(G)**.

Figures 6A,B showed that age was positively correlated with GRACE score or TIMI score (*P* < 0.05). Similarly, the positive relationship was found between age and IL-6 or IL-17 (Figures 6C,D, *P* < 0.05). On the contrary, age was negatively related to EPC migration, proliferation, and adhesion (Figures 6E–G, *P* < 0.05).

## Discussion

The present study firstly demonstrated that aging male NSTEMI patients had higher GRACE or TIMI score and lower EPC function than young. Circulating EPC function was significantly associated with the severity of NSTEMI. Moreover, aging male NSTEMI patients presented elevated IL-17 and IL-6 levels, which positively correlated with age and severity, and negatively connected with EPC function. These findings revealed the relationship between mitigated endothelial repairability and aging NSTEMI, with the latent involvement of systemic inflammation.

The GRACE and TIMI risk score have been recognized as useful indicators for evaluating disease severity and predicting adverse outcomes in NSTEMI (4, 5). Our results showed that GRACE and TIMI score of aging NSTEMI patients were higher than those of young, suggesting that age plays an important role in the prognosis of NSTEMI and it was similar with previous study which revealed the impact of age on the outcome of acute coronary syndrome (3). Interestingly, our research revealed that the correlation between age and GRACE score was stronger than that of TIMI score. Previous report showed that GRACE score was better than TIMI score in evaluating the long-term prognosis of NSTEMI (20). Together with our findings, it suggested that aging may have an important impact on the long-term prognosis of NSTEMI.

Circulating EPCs are critical for repairing endothelial damage and associated with prognosis of coronary artery diseases (21). Previous studies revealed that aging had close relationship with circulating EPC dysfunction (22, 23), and late-outgrowth EPC function significantly decreased in aging AMI (8). In the present study, we found that circulating EPC activity was significantly reduced in aging NSTEMI patients compared to young group and there was negative correlation between circulating EPC function and age. The results indicate that subdued endogenous endothelial repair capacity may be involved in the pathogenesis of aging NSTEMI.

In addition, our study found that circulating EPC function was significantly negative with GRACE or TIMI score in male patients with NSTEMI. To our knowledge, few studies had focused on the relationship between EPC function and GRACE or TIMI score. Considering the prediction value of GRACE score and TIMI score in NSTEMI (4, 5), it is reasonable to infer that circulating EFC function may be used as an indicator to assess the severity and prognosis of NSTEMI, and increment of circulating EPC function may be beneficial to the prognosis of NSTEMI.

Research illustrated that circulating levels of inflammatory cytokines are remarkably influenced by age (12). Consistent with previous findings, our study found that plasma levels of IL-6 and IL-17 significantly increased in aging NSTEMI and positively related with age. Moreover, it was reported that increment of pro-inflammatory cytokines might impair vascular damage, leading to EPC apoptosis and dysfunction (24, 25). In the present study, we found IL-6 and IL-17 were inversely correlated with EPC function in NSTEMI patients, suggesting the negative impact of inflammation on EPC function. Furthermore, our study showed that IL-6 and IL-17 were positively related to the severity of NSTEMI. A prior study pointed out that IL-6 was a positive feedback factor, which caused the aging mitochondrial dysfunction in coronary artery diseases (26). Systemic inflammation has been confirmed as an important risk factor in the pathogenesis of NSTEMI (27), while anti-inflammatory treatment is beneficial for the prognosis of coronary artery diseases (28). Thus, the current findings indicated that systemic inflammation may be the potential mechanism underlying hypofunction of circulating EPCs, which may be associated with adverse outcomes of aging NSTEMI.

The findings presented in this study have important clinical implications. First, circulating EPC function impaired in aging NSTEMI and inversely correlated with GRACE or TIMI score, implying that circulating EPC function may be an important biological indicator to provide prognostic information concerning aging NSTEMI. Interventions which may enhance EPC function may be helpful to improve the prognosis of NSTEMI, especially in aging male patients. Second, systemic inflammation may be partly responsible for the hypofunction of circulating EPCs and worse the prognosis of aging NSTEMI. Anti-inflammation treatment, such as IL-6 receptor antagonist tocilizumab or atorvastatin, may be beneficial to aging NSTEMI (28, 29).

Some limitations should be acknowledged. First, the present study does not follow up the cardiovascular outcomes in patients with NSTEMI. However, previous research had revealed the relationships between cardiovascular outcomes and GRACE or TIMI score (20), and our study illuminated the correlation between GRACE or TIMI score and EPC function which suggested the possible value of EPCs in predicting outcomes of NSTEMI. Further efforts will be made to verify the potential predictive value of circulating EPCs in NSTEMI. Second, we did not confirm the direct influence of systemic inflammation on EPC dysfunction. The *in vitro* experiment will be performed to discover the exact underlying mechanism.

## Conclusion

Our findings firstly uncovered that circulating EPC function decrement enhanced severity in aging male patients with NSTEMI, and systemic inflammation may be related to the alteration. The present study may provide us new insights into the underlying mechanism and therapeutic potentials for aging NSTEMI.

## Data Availability Statement

The original contributions presented in the study are included in the article/supplementary material, further inquiries can be directed to the corresponding author/s.

## Ethics Statement

The study was approved by the Ethics Committee of our hospital. The patients provided their written informed consent to participate in this study.

## Author Contributions

ZY designed the study. LZ, CZ, and CT did the experiments, analyzed the data, plotted the figures, and wrote the manuscript. YZ, BZ, ZL, and GL performed the experiments. All authors read and approved the final manuscript.

## Conflict of Interest

The authors declare that the research was conducted in the absence of any commercial or financial relationships that could be construed as a potential conflict of interest.
